# Complete supine percutaneous nephrolithotomy with GoPro^®^. Ten steps for success

**DOI:** 10.1590/S1677-5538.IBJU.2017.0337

**Published:** 2018

**Authors:** Fabio Carvalho Vicentini, Hugo Daniel Barone dos Santos, Carlos Alfredo Batagello, Julia Rothe Amundson, Evaristo Peixoto Oliveira, Giovanni Scala Marchini, Miguel Srougi, Willian Carlos Nahas, Eduardo Mazzucchi

**Affiliations:** 1 Divisão de Urologia, Grupo de Endourologia do Hospital das Clínicas, Faculdade de Medicina da Universidade de São Paulo, USP, São Paulo, SP, Brasil; 2 University of Miami, Miller School of Medicine, Miami, EUA

## Abstract

**Objective:**

To show a video of a complete supine Percutaneous Nephrolithotomy (csPCNL) performed for the treatment of a staghorn calculus, from the surgeon's point of view. The procedure was recorded with a GoPro^®^ camera, demonstrating the ten essential steps for a successful procedure.

**Materials and methods:**

The patient was a 38 years-old woman with 2.4cm of left kidney lower pole stone burden who presented with 3 months of lumbar pain and recurrent urinary tract infections. She had a previous diagnosis of polycystic kidney disease and chronic renal failure stage 2. CT scan showed two 1.2cm stones in the lower pole (Guy's Stone Score 2). She had a previous ipsilateral double J insertion due to an obstructive pyelonephritis. The csPCNL was uneventful with a single access in the lower pole. The surgeon had a Full HD GoPro Hero 4 Session^®^ camera mounted on his head, controlled by the surgical team with a remote control. All of the mains steps were recorded. Informed consent was obtained prior to the procedure.

**Results:**

The surgical time was 90 minutes. Hemoglobin drop was 0.5g/dL. A post-operative CT scan was stone-free. The patient was discharged 36 hours after surgery. The camera worked properly and didn't cause pain or muscle discomfort to the surgeon. The quality of the recorded movie was excellent.

**Conclusion:**

GoPro^®^ camera proved to be a very interesting tool to document surgeries without interfering with the procedure and with great educational potential. More studies should be conducted to evaluate the role of this equipment.

## ARTICLE INFO

**Available at: http://www.intbrazjurol.com.br/video-section/20170337_Vicentini_et_al**

**Int Braz J Urol. 2018; 44 (Video #13): 1046-1046**

